# Childhood Adversities Are Associated with Shorter Telomere Length at Adult Age both in Individuals with an Anxiety Disorder and Controls

**DOI:** 10.1371/journal.pone.0010826

**Published:** 2010-05-25

**Authors:** Laura Kananen, Ida Surakka, Sami Pirkola, Jaana Suvisaari, Jouko Lönnqvist, Leena Peltonen, Samuli Ripatti, Iiris Hovatta

**Affiliations:** 1 Research Program of Molecular Neurology, Faculty of Medicine, University of Helsinki, Helsinki, Finland; 2 Department of Medical Genetics, Faculty of Medicine, University of Helsinki, Helsinki, Finland; 3 Public Health Genomics Unit, National Institute for Health and Welfare, Helsinki, Finland; 4 FIMM, Institute of Molecular Medicine Finland, University of Helsinki, Helsinki, Finland; 5 Mental Health and Substance Abuse Services, National Institute for Health and Welfare, Helsinki, Finland; 6 Department of Psychiatry, Helsinki University Central Hospital, Helsinki, Finland; 7 The Broad Institute of MIT and Harvard, Cambridge, Massachusetts, United States of America; 8 Wellcome Trust Sanger Institute, Hinxton, Cambridge, United Kingdom; Leicester University, United Kingdom

## Abstract

Accelerated leukocyte telomere shortening has been previously associated to self-perceived stress and psychiatric disorders, including schizophrenia and mood disorders. We set out to investigate whether telomere length is affected in patients with anxiety disorders in which stress is a known risk factor. We also studied the effects of childhood and recent psychological distress on telomere length. We utilized samples from the nationally representative population-based Health 2000 Survey that was carried out between 2000–2001 in Finland to assess major public health problems and their determinants. We measured the relative telomere length of the peripheral blood cells by quantitative real-time PCR from 321 individuals with DSM-IV anxiety disorder or subthreshold diagnosis and 653 matched controls aged 30–87 years, who all had undergone the Composite International Diagnostic Interview. While telomere length did not differ significantly between cases and controls in the entire cohort, the older half of the anxiety disorder patients (48–87 years) exhibited significantly shorter telomeres than healthy controls of the same age (P = 0.013). Interestingly, shorter telomere length was also associated with a greater number of reported childhood adverse life events, among both the anxiety disorder cases and controls (P = 0.005). Childhood chronic or serious illness was the most significantly associated single event affecting telomere length at the adult age (P = 0.004). Self-reported current psychological distress did not affect telomere length. Our results suggest that childhood stress might lead to accelerated telomere shortening seen at the adult age. This finding has potentially important implications supporting the view that childhood adversities might have a considerable impact on well being later in life.

## Introduction

Telomeres consist of DNA repeats and associated proteins located at the ends of chromosomes. In highly proliferating cells, such as the germ and stem cells, telomere length is maintained by the telomerase enzyme, whereas in somatic cells, the activity of telomerase is low, leading to progressive telomere shortening with age, providing a marker for cellular aging [1], [2]. Leukocyte telomere length is a complex trait which is regulated by genetic [3]–[6] and environmental factors, such as smoking [7], and it has been associated with many disease phenotypes, such as cardiovascular diseases and diabetes [8]–[10].

An interest to telomere length in psychiatric phenotypes was awoken when shortened telomere length was associated to self-perceived stress [11], [12], and stress related to caregiving to Alzheimer's disease patients [13]. In addition to stress at the adult age, childhood stress might affect telomere length later in life since childhood maltreatment was recently associated with telomere shortening in 31 psychiatrically healthy adults [14]. The hypothesis that stress affects telomere length is further supported by animal experiments, as exposing the offspring of wild-caught mice to stressful conditions led to telomere attrition [15]. Furthermore, negative expectations for the future seem to increase telomere attrition as pessimism was correlated with leukocyte telomere length and elevated interleukin-6 levels in a recent study [16]. Moreover, decreased perceived mental health was associated with shorter leukocyte telomere length in patients with chronic heart failure [17]. Regarding psychiatric disorders, shortened telomere length has been observed in mood disorders [18] and schizophrenia [19], [20] suggesting a putative mechanism explaining why many psychiatric disorders lead to increased susceptibility to somatic illness [11].

Anxiety disorders are complex diseases with both genetic and environmental susceptibility factors. Despite the identification of several predisposing genes [21] and environmental factors, such as stressful life events and adverse childhood experiences [22]–[26], the specific disease mechanisms remain largely unknown. We hypothesized that accelerated telomere shortening might be involved in the etiology of anxiety disorders, and set out to test this hypothesis in anxiety disorder patients and matched controls, spanning 57 years of age and drawn from a randomly selected population-based Health 2000 cohort (www.terveys2000.fi). We additionally hypothesized that advanced age might be needed in order for stress to have had an effect on telomere shortening and therefore, we divided the sample by the median age (48 years), and analyzed these two age groups also separately. In addition, we investigated the effect of childhood adverse events and recent psychological distress on telomere length within the same sample. These both have been reported to associate with current DSM-IV mental disorders in previous studies of the Health 2000 project [27], [28]. Our results show that there was no difference in the telomere length between the anxiety disorder patients and controls in the entire cohort, although older anxiety disorder patients may have accelerated telomere shortening. Recent psychological distress did not have an effect on the telomere length, but the number of childhood adverse life events correlated significantly with shorter telomere length both in cases and controls.

## Results

### Telomere length in anxiety disorders

**Figure 1:**
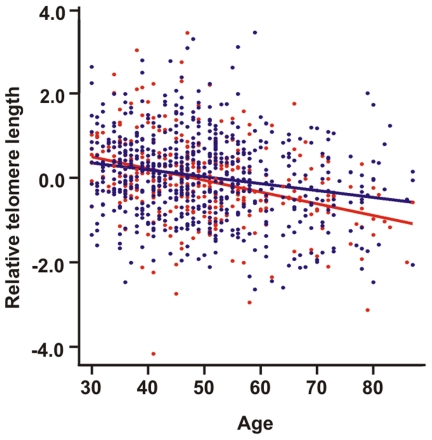
Telomere length as a function of age. Anxiety disorder core and subthreshold cases (N = 321) are shown with red dots and controls (N = 653) with blue dots, each dot representing one individual. Regression lines for both groups are shown with the same color coding.

Anxiety disorder cases and two matched controls per case were identified from the Health 2000 epidemiological cohort, which is a randomly selected sample of the Finnish population. We measured the relative telomere length from peripheral blood leucocytes of the participants using a qPCR-based method [29], in which the signal value from a qPCR reaction with telomere sequence specific primers was divided by the signal value from a qPCR reaction with beta-hemoglobin gene specific primers (single copy control gene). After quality control, our study sample consisted of 939 individuals between 30 and 87 years of age (Table 1). Figure 1 shows the telomere length as a function of age for anxiety disorder cases and controls. As expected, age was significantly associated with telomere length (P = 4×10^−16^) in a linear regression analysis, as telomeres shorten during aging. There was no significant difference in telomere length between sexes (P = 0.154) or between cases and controls (Table 2).

**Table 1:** The Health 2000 anxiety disorder sample analyzed in this study.

Characteristic	Core cases^1^	Subthreshold cases^2^	Core and subthreshold cases	Controls
**Number of individuals**	282	39	321	653
**Age**	49.82 (±12.74)^3^	49.03 (±12.97)	49.7 (±12.8)	49.8 (±12.6)
**Women**	61.7%	71.8%	62.9%	63.5%
**GHQ-12 score** 4	5.45 (±4.18)	4.92 (±4.40)	5.39 (±4.21)	1.14 (±2.41)
**Number of childhood adverse life events**	1.92 (±1.82)	2.10 (±2.00)	1.94 (±1.83)	0.97 (±1.28)

1 Cases meeting DSM-IV anxiety disorder diagnosis.

2 Cases meeting DMS-IV subthreshold anxiety disorder diagnosis.

3 Mean ± standard deviation.

4 Sum score of the 12-item General Health Questionnaire.

**Table 2:** Telomere length is affected by childhood adverse life events but not by anxiety disorder diagnosis or recent psychological stress.

Regression model	β^1^	se^2^	P-value
**Anxiety disorder**	−0.080	0.069	0.224
**GHQ-12 score** 3	−0.002	0.009	0.838
**Number of childhood adversities** 4	−0.090	0.032	**0.005**

1 Difference in standardized telomere length for one unit or category change in each independent variable.

2 Standard error of the mean.

3 Sum score of the 12-item General Health Questionnaire.

4 Categorized to 0 adversities, 1 adversity, 2 or 3 adversities, and 4 or more adversities.

Results from three independent regression models are shown in which sex and age adjusted telomere length was explained by either anxiety disorder status, GHQ-12 score, or number of childhood adversities.

We then analyzed the two age groups separately. There was no significant difference in the telomere length between cases and controls in the younger age group (30–47 years of age). However, in the older age group (48–87 years of age), the telomere length was significantly shorter in anxiety disorder cases compared to controls (β = −0.240, se = 0.096, P = 0.013).

### Current stress and telomere length

We examined the role of current psychological distress, as measured by the 12 questions of the General Health Questionnaire (GHQ-12), on telomere length. Even though the GHQ-12 sum score correlated significantly with the presence of a DSM-IV anxiety disorder or subthreshold diagnosis (χ^2^ = 307.357, df = 12, P<1×10^−16^), it did not have an effect on telomere length (Table 2).

### Childhood stress and telomere length

**Figure 2:**
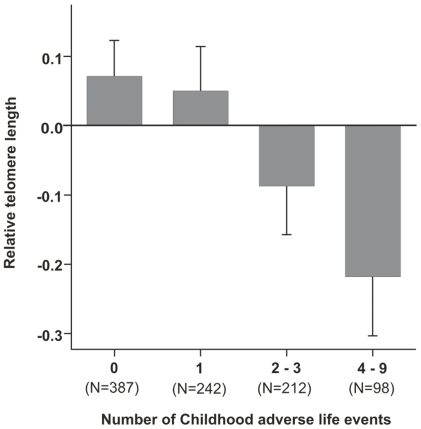
The number of childhood adverse life events affects relative telomere length at adult age. Relative telomere length is adjusted for age and sex. Each bar presents group mean (± standard error of the mean).

We next investigated the number of childhood adverse life events as a measure of childhood distress. The number of childhood adverse life events correlated significantly with the anxiety disorder diagnosis (χ^2^ = 74.087, df = 3, P = 5.69×10^−14^). We observed a significant effect of the number of childhood adversities on the age and sex adjusted telomere length (Table 2, Figure 2). In order to further explore the specific individual childhood adversities, we analyzed their effect on telomere length individually. Parental unemployment (β = −0.157, se = 0.059, P = 0.008) and own chronic or serious illness (β = −0.345, se = 0.088, P = 0.0001) showed independent effects, also when modeled jointly. The effect of the own chronic or serious illness on telomere length is significant even after correcting for multiple testing for the number of adversities using the Bonferroni method.

### Effect of other telomere length-associated factors

Telomere length might be affected by several biological and lifestyle factors, including body mass index (BMI) [7], [30], [31], smoking [7], [32], homocysteine levels [33], cardiovascular disease risk factors [8], [9], [34]–[37], insulin levels or resistance [10], [38], [39], diagnosis of diabetes [40], [41], sleep duration [16], or physical inactivity [42]. In addition, factors related to the anxiety disorder diagnosis, such as medication or other comorbid illnesses, might have an influence on telomere length. Therefore, we carried out additional analyses to examine the relationship between the number of adverse childhood life events, telomere length, and putative confounders.

Since our cohort is ascertained through anxiety disorder diagnosis, and 28% of the cases have a comorbid major depressive disorder and 22% a comorbid alcohol use disorder, we modeled these factors, the number of childhood adverse life events, and the telomere length jointly. Only the effect of the number of childhood adverse life events on telomere length was statistically significant (β = −0.091, se = 0.032, P = 0.005).

We obtained information concerning the recent psychiatric medication, and studied the effect of benzodiazepine, selective serotonin reuptake inhibitor, tricyclic antidepressant, other antidepressant, antipsychotic, and sleep medication on telomere length together with the number of adverse life events. Again, only the number of childhood adverse life events had a significant effect on telomere length (β = −0.082, se = 0.032, P = 0.009).

Risk factors for cardiovascular diseases have been shown to have an effect on telomere length in several studies. We analyzed the effect of BMI, systolic and diastolic blood pressure, homocysteine levels, and triglyceride, high-density lipoprotein (HDL) and low-density lipoprotein (LDL) levels on telomere length together with the number of childhood adverse life events. In this analysis as well, only the number of childhood adverse life events had a significant effect on telomere length (β = −0.079, se = 0.031, P = 0.012).

Diabetes and related risk factors have also been associated with shorter telomere length in many studies. In a model consisting of the number of childhood adverse life events, diagnosis of diabetes, BMI, and blood glucose and insulin levels, the number of childhood adverse life events was the only factor explaining the shorter telomere length (β = −0.085, se = 0.031, P = 0.006).

In the last model, we examined life style factors, including physical activity, smoking, and duration of sleep together with childhood adversities. While the number of childhood adverse life events had a significant effect on telomere length (β = −0.064, se = 0.032, P = 0.043), so did smoking (β = −0.181, se = 0.081, P = 0.018) and duration of sleep (β = −0.056, se = 0.027, P = 0.037).

### Telomere length and sociodemographic factors

In a linear regression model including sociodemographic factors (age, sex, hospital district, marital status, education, and current employment status), age was the only significant explaining factor of the telomere length (data not shown), suggesting that other sociodemographic factors do not have an effect on telomere length in this sample.

## Discussion

We have observed a correlation between shorter telomere length at adult age and the reported number of childhood adverse life events in a subsample of an epidemiological cohort consisting of anxiety disorder patients and matched controls. Interestingly, this finding was not dependent on the anxiety disorder status, although anxiety disorder patients had a larger number of childhood adversities compared to the controls. The most significantly associated single adversity was childhood chronic or severe illness which may affect cellular ageing through yet unknown direct physiological and indirect psychological mechanisms. The reported childhood adversities may represent a lifelong course of stress factors, partly traumatic in origin and later complicated by temporally secondary mental disorders [27]. Our results can be considered as a partial replication of a recently published study [14] in which childhood maltreatment was associated with shorter telomere length at the adult age in 31 individuals with no psychiatric disorders, although different measures of childhood adversity was used.

Our sample derives from the nationwide Health 2000 Study which was carried out to assess welfare of Finnish people and which represents the entire population over 30 years of age [28]. Therefore, we were able to control for a large number of possible confounders, which is typically not possible in a clinic-based setting. In addition to childhood adversities, smoking and duration of sleep affected telomere length. Smoking decreased telomere length while the duration of sleep correlated positively with it. The adjustment of telomere length by these two factors decreased the β-value of the number of adverse life events by about one third, suggesting that this proportion of the effect may be explained by these two life-style factors. Smoking has been shown to affect telomere length in numerous studies e.g. [7], [32], but duration of sleep has not, although some earlier studies did adjust telomere length by sleep duration [16].

Interestingly, sum score of GHQ, which measures recent psychological distress, correlated significantly with the anxiety disorder status, but did not have an effect on telomere length. Several studies have observed a correlation of shorter telomere length with current psychological stress [11], [12] or more chronic stress [13], although a negative report exists as well [14]. So far, our study is the only population-based cohort to investigate the effect of current psychological stress with telomere length, and the instruments to measure stress have varied across studies.

Our initial hypothesis was that anxiety disorder patients would have shorter telomere length compared to controls. However, telomere length did not differ between anxiety disorder patients and controls in the entire sample including individuals 30–87 years of age. In the older half of the patients (48 years or older), we did observe shorter telomere length in cases compared to controls. This finding might indicate that prolonged stress related to having an anxiety disorder might be needed for accelerated telomere shortening, and this hypothesis should be tested in other anxiety disorder cohorts.

Although this population-based cohort enabled us to investigate the relationship of telomere length and anxiety disorders in light of a multitude of confounding factors, it also has some limitations. The specimen available to us was DNA samples extracted from peripheral blood. Because we did not have access to blood samples, we were limited to only investigate the relative telomere length, and could not assay telomerase enzyme activity or oxidative stress markers, factors that have earlier been associated with perceived stress. Furthermore, the CIDI interview was conducted to detect present (i.e. past 12-month) anxiety disorder diagnoses, and therefore we might have missed some patients in remission. In addition, it would have been interesting to study the effect of long-term stress on the telomere length, but this was not possible in our cohort. However, the correlation of the number of childhood adverse life events and the GHQ sum score was highly significant (Spearman R = 0.279, two-tailed P = 5.28×10^−18^), suggesting a relationship between childhood stress and recent psychological distress.

Mechanisms that contribute to accelerated telomere shortening in vivo remain largely unknown. In vitro, oxidative stress has been shown to shorten telomeres, and antioxidants to reverse the accelerated telomere shortening induced by increased oxidative stress [2]. Supporting this hypothesis, Epel et al. [11] observed higher oxidative stress and shorter telomere length with chronic psychological stress. In the same sample of 62 healthy women, telomere length was associated to elevated stress hormone levels, and telomerase enzyme activity to cardiovascular disease risk factors [43]. Similarly, increased oxidative stress correlated with shorter telomere length in a study of insulin resistance and hypertension [39]. Moreover, it has been suggested that telomere length might serve as a biomarker of cumulative exposure to oxidative stress and a prognostic indicator for risk of late-life diseases [2]. The hypothesis that white blood cell telomere length is associated to morbidity or mortality has recently been tested, but the results have been conflicting [44]–[47]. Interestingly, it has been shown that maternal diet influences the aortic telomere length through changes in DNA single stranded breaks, antioxidant capacity, and oxidative stress in rat pups [48]. Oxidative stress has been suggested to play a role in the etiology of anxiety disorders, and support for the involvement of oxidative stress in the regulation of anxiety-like behavior in rodents has been observed in several studies [49]–[53].

Shorter telomere length has been observed in other psychiatric diseases, including mood disorders [18] and schizophrenia [19], and in schizophrenia patients with poor treatment response [20]. Importantly, oxidative stress has been shown to be involved in the etiology of these disorders [54], [55]. Another intriguing putative mechanism involves adult neurogenesis, which has been shown to be involved in mental health and illness [56]. It was recently shown that deficient neurogenesis correlates with reductions in telomere length in adult subependymal zone neural stem cells [57].

Due to the lack of longitudinal studies, it is not known whether telomere shortening is a cause or a consequence of stress. A recent work in mice suggests that stress over a period of six months leads to accelerated telomere shortening in stressed, but not in the control mice, and even increased telomere length was observed in some control groups [15]. In humans, telomere lengthening has been observed in a subset of individuals in two epidemiological studies in which samples were taken approximately 10 years apart [58], [59]. In both studies the telomere length at the follow-up was proportional to the telomere length at the baseline, with those individuals with the shortest telomeres to start with showing no difference or increase in the telomere length. Interestingly, it has also been suggested that a comprehensive lifestyle change might increase telomerase activity, a potential precursor to telomere length, [60] putatively leading to telomere lengthening over time as a result of healthier habits and less psychological and physiological stress, counteracting the influence of a chronic disease on telomere attrition. Therefore, it might be possible to affect the telomere length of those individuals with short telomeres due to childhood physiological or psychological stress, either by life-style choices or by pharmacological means to be developed. For this reason further studies on the mechanisms linking stress and telomere length are needed.

## Materials and Methods

### Ethics statement

This study was approved by the ethics committee of the National Public Health Institute and written informed consent was obtained from all participants.

### Study sample

Subjects of this study derive from the population-based epidemiological Health 2000 cohort collected by the National Public Health Institute of Finland (since 1 January 2009 the National Institute for Health and Welfare) during 2000 and 2001. The cohort was collected to assess the major public health problems, functioning and their determinants of the adult Finns, aged 30 years or older [61]. Therefore, we have detailed information concerning the well-being and lifestyle of the participants. Altogether 6005 individuals participated in a structured psychiatric interview (Munich Composite International Diagnostic Interview, M-CIDI) to diagnose current (past 12 months) mental illness using the DSM-IV criteria [62]. The anxiety disorders that were diagnosed with the version of M-CIDI used in this study include panic disorder, generalized anxiety disorder, social phobia, agoraphobia, and phobia not otherwise specified. In addition to the individuals meeting the DSM-IV criteria for these anxiety disorders, the M-CIDI interview enabled us to identify so called subthreshold cases who do not meet all full diagnostic criteria, but likely share the same genetic liability and were included to increase the sample size. The subthreshold cases fulfil most screen and core symptoms of the specific diagnoses but the number of additional symptoms may not have added up to the full DSM-IV criteria. Similar approaches using extended diagnostic criteria have been used in both epidemiological and genetic studies of anxiety disorders [63]–[65]. The number of cases in each category was: 108 core panic disorder cases, 73 core and 30 subthreshold generalized anxiety disorder cases, 58 core and 7 subthreshold social phobia cases, 31 core and 15 subthreshold agoraphobia cases, and 58 core cases of phobia, not otherwise specified. We then selected two control individuals for each case, matched by sex, age (+/−1 year), and university hospital district (N = 5 in the entire country, each with approximately 1 million inhabitants). The final sample of individuals with DNA available included 321 cases and 653 controls. A comorbid other anxiety disorder was present in 16%, a comorbid major depressive disorder or dysthymia in 28% and a comorbid alcohol use disorder in 22% of the cases, as described earlier in more detail [28], [66], [67]. We also used the available information concerning the current use of psychiatric medication based on self-report. Current medication was asked about in the home interview, and in most cases registered based on prescriptions shown to the interviewer.

In addition to the anxiety-related variables, we analyzed a large number of other variables available as part of the epidemiological study [61], [68]. Sociodemographic factors included age, sex, hospital district as described above, marital status (1. married or living as married, 2. divorced, 3. widow, 4. unmarried), education (divided into three categories based on information on general education and on higher and vocational education), and current employment status (1. full or part-time employed, 2. unemployed or laid off, 3. retired, 4. other). Mental health related variables (based on DSM-IV criteria in M-CIDI interview) included major depressive disorder and/or dysthymia (grouped together as depressive disorders), as well as diagnosis of alcohol abuse or alcohol dependence (grouped together as alcohol use disorders).

Since self-perceived stress has been associated to accelerated telomere shortening, the 12-item GHQ (General Health Questionnaire) was used to assess whether reported current psychological distress has an impact on telomere shortening. Childhood adverse life events have been shown to be a significant risk factor for anxiety disorders in this cohort [27]. The questionnaires given to the subjects during a home interview contained a series of 11 questions about their childhood social environment. The subjects were instructed to choose “no”, “yes”, or “cannot say” when asked the following: “When you think about your growth years, i.e., before you were aged 16, …”

1. Did your family have long-term financial difficulties?
2. Was your father or mother often unemployed although they wanted to work?
3. Did your father or mother suffer from some serious disease or disability?
4. Did your father have alcohol problems?
5. Did your mother have alcohol problems?
6. Did your father have any mental health problem, e.g., schizophrenia, other psychosis, or depression?
7. Did your mother have any mental health problem, e.g., schizophrenia, other psychosis, or depression?
8. Were there any serious conflicts within your family?
9. Did your parents divorce?
10. Were you yourself seriously or chronically ill?
11. Were you bullied at school?

Only “yes” answers were coded positive, and the total number of reported adversities per subject was recorded from 0 to 11. In our sample, the highest observed sum score was 9. Cronbach's alpha, which measures the relationship between individual adversity items, was 0.67 in our anxiety disorder subsample of the Health 2000 cohort (N = 974), and 0.60 among all Health 2000 participants (N = 8028), suggesting that although there is some correlation between the responses to the individual items, the correlation is relatively low and therefore justifies the inspection of individual questions one by one. We studied the effect of the number of possibly stressful childhood adversities on telomere length by categorizing the sum score to four groups: 0 adversities, 1 adversity, 2 or 3 adversities, and 4 or more adversities, in addition to studying them one at a time.

In addition, we obtained information concerning other factors that might affect telomere length including BMI, smoking (yes or no), homocysteine levels, HDL and LDL cholesterol, triglyceride levels, diastolic and systolic blood pressure, glucose levels, insulin levels, diagnosis of diabetes, sleeping (hours per night on average), and physical activity (1. ideal = at least 30 min. leisure time physical activity at least 4 days a week and at least 30 min. walking or bicycling to work, 2. adequate = at least 30 min. leisure time physical activity at least 4 days a week or at least 30 min. walking or bicycling to work, 3. uncertain, 4. inadequate).

### Telomere length measurement

Telomere length was determined from genomic DNA extracted from peripheral blood by a quantitative real-time PCR-based method [19], [29] with modifications as follows. Relative telomere length was determined by comparing the value from absolute quantification of telomere DNA with a single copy reference gene, ß-hemoglobin (T/S ratio). These two assays were carried out as separate reactions on separate plates maintaining the sample positions between the two plates. Amplification signals were quantified by the standard curve method using a DNA template series (1.0, 2.0, 5.0, 10, 20, 40, and 60 ng) on every plate. All randomized DNA samples (15 ng) and standard dilutions were processed as triplicates onto 384 well plates using a Hydra 96 robot (Robbins Scientific, Sunnyvale, CA, USA), and dried for 24 h at +37°C. Specific reaction mix for telomere reaction included 270 nM tel1b primer (5′-CGGTTT(GTTTGG)5GTT-3′) and 900 nM tel2b primer (5′-GGCTTG(CCTTAC)5CCT-3′), 150 nM ROX (Invitrogen, Carlsbad, CA, USA), 0.2× SYBR Green I (Invitrogen, Carlsbad, CA, USA), 5 mM DTT (Sigma-Aldrich, Saint Louis, MO, USA), 1% DMSO (Sigma-Aldrich, Saint Louis, MO, USA), 0.2 mM of each dNTP (Fermentas International, Burlington, Canada), and 1.25 U AmpliTaq Gold DNA polymerase (Applied Biosystems, Foster City, CA, USA) in a total volume of 15 µl AmpliTaq Gold Buffer I. Single copy gene reaction mix included 300 nM Hgb1 primer (5′-GCTTCTGACACAACTGTGTTCACTAGC-3′) and Hgb2 primer (5′-CACCAACTTCATCCACGTTCACC-3′) in a total volume of 15 µl of Dynamo HS SYBR Green qPCR mix (Finnzymes, Espoo, Finland). The cycling conditions for telomere amplification were: 10 minutes at 95°C followed by 25 cycles at 95°C for 15 s and 54°C for 2 min with signal acquisition. The cycling conditions for single copy gene amplification were: 95°C for 10 min followed by 35 cycles at 95°C for 15 s, 58°C for 20 s, 72°C for 20 s with signal acquisition. These procedures were carried out with an ABI PRISM 7900 HT qPCR machine (Applied Biosystems, Foster City, CA, USA). Altogether 11 plates were analyzed with telomere specific primers and 11 plates with ß-hemoglobin specific primers.

We performed rigorous quality control to ensure data quality. Samples with standard deviation of >0.5 between triplicates were omitted from the analysis (N = 25). We monitored the correlation coefficient of the standard curves and it was 0.995 on average for the telomere reaction (range 0.987 to 0.999) and 0.997 on average for the ß-hemoglobin reaction (range 0.995 to 0.999). The efficiency of the PCR reaction can be estimated from the slope of the standard curve, and it was 97.6% on average for the telomere reaction (range 79.9 to 116.6%) and 100.5% on average for the ß-hemoglobin reaction (range 92.0 to 107.2%). In addition, the qPCR plates included a short and a normal telomere length control DNA samples. The relative normalized signal intensity of the short telomere control was on average 0.23 (range 0.18 to 0.28) and 1.17 (range 1.03 to 1.39) on average for the normal telomere length control. Furthermore, we performed a melting curve analysis at the end of each reaction to verify specific PCR amplification.

### Statistical analysis

Because the T/S ratios were not normally distributed, we first transformed these values using the natural logarithm. At this point, outlier samples (N = 10) were removed. We then adjusted for batch to batch variation, and standardized the T/S ratio values by plate to the mean of 0 and to the standard deviation of 1.

Statistical analyses were performed using the statistical software SPSS version 15.0. We confirmed that all assumptions were met prior to analyses, including examination of normal distribution graphically using histograms and testing of equal variances with Levene's test of the standardized telomere length data in cases and controls.

Multiple linear regression modeling was used with the transformed and standardized telomere length as the dependent variable to assess the influence of anxiety disorders, current stress, childhood stress, and various covariates on telomere length. Age and sex were adjusted in regression models. Nominal significance level was set to p<0.05.

Telomere length and the number of childhood adversities were visualized using the SPSS version 15.0.
